# Remarks on the Treatment of Gleet by Local Remedies

**Published:** 1859-02

**Authors:** 


### *Remarks on the Treatment of Gleet by Local Remedies.*

By Wm. Otterson, M. D., of Brooklyn, N. Y.

The slight discharge from the urethra so often met with after an attack of gonorrhœa, and variously known as "chronic blennorrhagia, gleet," etc., occurs under various circumstances, and its seat, cause, and treatment, are differently given by different authors. Cooper, Vidal, Acton, Hunter, Chelius, etc., give different views of the seat and pathology of the disease; yet they all concur in the general plan of treatment, viz., stimulating and astringent injections, cubebs, terebinthinates, and speak, as a last resort, of sometimes using the bougie. We find these writers placing the seat of the disease in the prostate (Cowper's) glands, the lining membrane of the urethra, the lacunæ, and in the fossa navicularis. I was disposed to think that Sir A. Cooper was nearest right, and that the glans penis and the lacunæ therein are the real seat of this affection. What the precise pathological condition of the glans is, I am unable to say; but it would appear that owing to its delicate structure, and the fact of its being the most frequent seat of acute attacks, that the onus of the chronic form of the disease should be centered upon this portion of the organ; and that many of the most obstinate cases of gleet (in the absence of stricture and disease of the prostate) are dependent upon a morbid condition of the glans, and not upon any disease or change of the lining membrane of the urethra itself. My reasons for this belief are: 1st, the amount and character of the discharge; 2d, the point of irritability upon passing an instrument; 3d, the inadequacy of injections to control the disease permanently; 4th, the failure of internal medication or hygiene to exert any special influence over it.

1st. The discharge is found to be nothing more than opaque mucus, which may vary in consistence according to circumstances. This in the morning occupies the meatus, at times almost blocking it up, and is easily removed if the patient runs the finger along the under surface of the urethra, beginning about an inch from the meatus, when the secretion escapes; after that, the urethra may be squeezed from the prostatic region to the meatus, and yet no more of the discharge be procured.

2d. In passing an instrument, when about an inch from the meatus, and corresponding with the fossa naviculatis, the patient complains of pain, and after the instrument passes this point the whole tract of the urethra beyond is free from any irritability. This I have found as an almost invariable result in my cases of obstinate and long standing gleet, when there was no stricture.

3d. Injections of all kinds have utterly failed to make any permanent impression upon the discharge, though there have been remissions for a few days at different times.

4. Internal remedies have failed to exert any perceptible influence over it.

If the lining membrane of the urethra were alone involved, the result of medication would be different, for we all know that, in acute gonorrhœa, many of what are called specific medicines *do* exert a modifying and decidedly beneficial influence over the diseased tract of the canal. But in this form of gleet they fail, because their action is not adapted to the disease of this particular structure. If Cowper's glands are the seat, then the application the author makes in these cases would appear to be useless, as the remedies are not applied to the portion of the canal where they are situated.

*Treatment.* I first examine the patient to ascertain if there is stricture, narrowing of the canal, etc., and if there are any such obstructions the usual measures are adopted to overcome them. Sometimes this alone will effect a cure; but if the discharge continues, I introduce a No. 8 metallic grooved bougie, the groove being filled either with citrine ointment or calomel; the latter I prefer as being more certain in its action. The instrument need be passed only a sufficient length up the urethra to get the point beyond the glans penis, when it should be carefully revolved several times; it should be allowed to remain in for three or five minutes, and then a probe, adapted to the curve of the instrument, should be passed down the groove; if they are then withdrawn simultaneously the whole of the calomel will be left in apposition with the parts.

This may be repeated two or three times a week, at the same time that the bowels are kept soluble. Upon withdrawing the instrument a little smarting may be complained of, and perhaps a few drops of blood follow, but this is not important. If this plan of treatment is followed assiduously, you can assure your patient with much confidence, that a very short time only will be required to remove every trace of the most obstinate and hitherto intractable gleet.

The writer has had quite a large number of these cases under his care, and formerly was in the habit of adopting the stereotyped practice, and have them linger from month to month, and finally to pass into other hands; but since adopting the view that *benefit can only be derived by direct alterative medication to the seat of the disease*, and pursuing the plan of treatment laid down in this paper, he has not failed to cure a single case, marked benefit arising after three or four applications even in the most unfavorable and long standing cases.

As some authors have recommended the use of mercury, with a view to produce its specific effect upon the system, I transfer one case from my notebook, illustrating its action in this affection.

Case I. L. W. consulted me an account of a chancre, which I cauterized with solid nit. argent., and after giving a cathartic, kept him upon blue mass and opium, until its specific action became manifest, and as there was some induration, kept up a slight mercurial action for some time by hyd. potass. and ext. sarsp. comp. This treatment being continued about two weeks, the chancre healed kindly, when, for the first time, the patient informed me that he had been suffering from gleet for about eight months, which, at the time he first applied, was quite severe; but he, thinking that the medication and regimen adopted for the cure of his syphilis, would also suffice for his gleet, did not mention it till after his primary disease was cured; but finding he was not free from his old discharge he wished further advice. I proposed the bougie, but he having been present when I had introdnced it on his friend, desired that some other measure should first be tried. I consented, after giving an unfavorable prognosis. The old routine of injections, etc., was gone through, and after one month's trial, he concluded to submit to the bougie. Upon examination, no irritable point was found after the point of the instrument passed the fossa navicularis, no stricture or narrowing of the urethra. A perfect cure was effected in about three weeks, no relapse following.

CASE II. J. D. applied to me on account of a gleet which had troubled him about five months, the sequel of a gonorrhœa which he had treated himself with injections and bals. copab. Upon examination, I found some narrowing of the canal just anterior to the bulb, with irritability at this point, and at the point corresponding with the seat of the fossa navicularis. This constriction readily gave way by the use of graduated bougie, but the gleet still continued. I treated him on the old plan for six months, during which time all the different remedies were tried, and strict rules of regimen enjoined. In this time there were several remissions, but nothing permanent. At last I introduced the medicated bougie twice a week, at the same time removing entirely my dietetical restrictions. At the end of six weeks, although he daily drank large quantities of beer, he was entirely cured. Some may be disposed to think that this case required tonics and nourishing diet, but these had been given as one of the changes during the six months of the old treatment.—*New York Journal of Medicine.*

